# FcγRIIIa receptor polymorphism influences NK cell mediated ADCC activity against HIV

**DOI:** 10.1186/s12879-019-4674-z

**Published:** 2019-12-16

**Authors:** Sneha Pramod Talathi, Nawaj Najir Shaikh, Sudhanshu Shekhar Pandey, Vandana Ashish Saxena, Megha Sunil Mamulwar, Madhuri Rajeev Thakar

**Affiliations:** 1 Department of Immunology, National AIDS Research Institute, Plot No. 73, G-Block, MIDC, Bhosari, Pune, Maharashtra 411026 India; 2 Department of Epidemiology, National AIDS Research Institute, Pune, 411026 India

**Keywords:** FcγRIIIa-V176F polymorphism, FcγRIIIa-Y158H polymorphism, NK cells, ADCC, HIV

## Abstract

**Background:**

HIV-specific Antibody Dependent Cell Cytotoxicity (ADCC) has shown to be important in HIV control and resistance. The ADCC is mediated primarily by natural killer cell activated through the binding of FcγRIIIa receptor to the Fc portion of antibody bound to the antigen expressed on the infected cells. However, no data is available on the influence of the polymorphism in FcγRIIIa receptor on HIV-specific ADCC response.

**Methods:**

The Sanger’s method of sequencing was used to sequence the exon of FcγRIIIa receptor while the ADCC activity was determined using NK cell activation assay. The polymorphism in FcγRIIIa receptor was assessed in HIV-infected Indian individuals with or without HIV-specific ADCC antibodies and its influence on the magnitude of HIV-specific ADCC responses was analyzed.

**Results:**

Two polymorphisms: V176F (rs396991) and Y158H (rs396716) were observed. The Y158H polymorphism is reported for the first time in Indian population. Both, V176F (V/V genotype) (*p* = 0.004) and Y158H (Y/H genotype) (*p* = 0.032) were found to be significantly associated with higher magnitude of HIV-specific ADCC response.

**Conclusion:**

The study underscores the role of polymorphism in the FcγRIIIa receptor on HIV-specific ADCC response and suggests that the screening of the individuals for FcγRIIIa-V176F and Y158H polymorphisms could be useful for prediction of efficient treatment in monoclonal antibody-based therapies aimed at ADCC in HIV infection.

## Background

Antibody dependent cell mediated cytotoxicity (ADCC) has shown to be associated with slower disease progression in Human Immunodeficiency Virus (HIV) infection [1–5]. The RV144 HIV vaccine trial has shown the correlation between lower infection rates and higher ADCC responses among vaccine recipients [6], highlighting the importance of ADCC responses in HIV protection. The ADCC is mediated primarily by natural killer (NK) cells through binding of the FcγRIIIa (CD16a) receptor with the Fc portion of the antibodies bound to the specific antigen expressed on the target cells. This binding initiates secretion of granzyme and perforin by NK cells resulting in the lysis of the target cell [7]. Thus, for strong ADCC responses; binding affinity of FcγRIIIa receptor and the Fc portion of the antibody is important [8, 9] and the polymorphic changes in this receptor are likely to affect the binding affinity of the FcγRIIIa receptor which might influence the ADCC activity [10–12]. Single nucleotide polymorphism (SNP) of Fc receptor gene of FcγRIIIa - V158F or V176F is known to result in low functioning receptor with phenylalanine (F) and high functioning receptor with valine (V) [13, 14]. Populations like Dutch, [15] Sami, Norwegian, [16] Japanese, [14] African American, [12] British, and northern Indian [17] have shown this polymorphism at 158 amino acid position while Asian Indian [13], Malaysian [5], Chinese [18], Korean [19], Thais [20] etc. have shown this polymorphism at 176 position. The V158F polymorphism was found to be associated with augmented ADCC response to rituximab, an anti-cancer monoclonal therapeutic antibody in in-vitro experiments, suggesting that the individuals expressing at least one valine at amino acid position 158 in FcγRIIIa might show better rituximab-mediated ADCC [14, 21]. This polymorphism is in the exon 4 and is reported to alter the protein confirmation and thus influences binding affinity of FcγRIIIa and Fc region of antibodies involved in ADCC [14, 21]. However, no information is available about this polymorphism in HIV infection and about its influence on the HIV-specific ADCC responses. Therefore in the present study, the presence of polymorphism in FcγRIIIa receptor was assessed by sequencing the exon 4 of FcγRIIIa receptor and the association of this polymorphism with the HIV-specific ADCC responses was determined. Our study population showed F to V transition at 176 amino acid position when the sequences were compared with the reference sequence from UCSC browser. Additionally, another polymorphism was observed at amino acid position 158, resulting in a substitution of tyrosine to histidine (Y158H). Both the polymorphisms observed to influence HIV-specific ADCC activity. Additionally Y158H has shown possibility of association with HIV acquisition.

## Methods

### Study participants

Study participants were identified from individuals followed up regularly at outpatient clinics of National AIDS Research Institute. Sixty-three HIV infected (42 female/21 male) ART naive individuals at various stages of HIV disease and 67 HIV uninfected healthy controls (HCs) (26 female/41 male) were enrolled in the study.

### ADCC (NK cell activation) assay

**Fig. 1:**
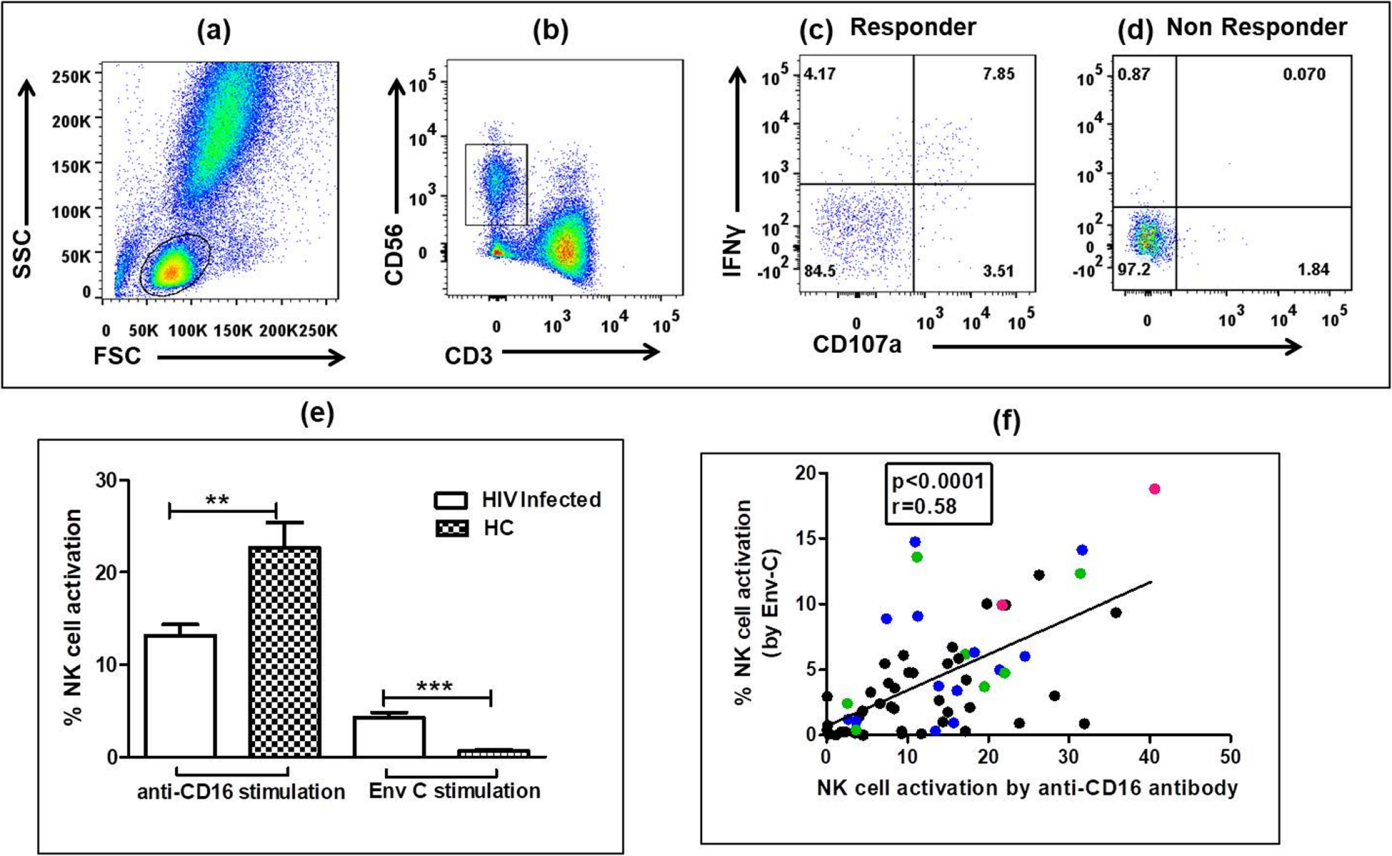
Anti-HIV ADCC measured by NK cell activation (the sum of the percentage of activated NK cells, expressing only CD107a or IFN-γ, or both CD107a and IFN-γ) in study participants. a-d: Flow cytometry plots showing the gating strategy used **a** Lymphocytes were gated based on FSC and SSC and **b** NK cells were identified as CD3-CD56+ cells and were assessed for CD107a or IFNγ expression or dual expression. Figures c and d shows representative plots showing NK cell activation observed in **c** HIV-1 Env-C responder and **d** HIV-1 Env-C non-responder **e** Bar graph shows % NK cell activation by anti-CD16 antibody and HIV-1 Env-C stimulation in HIV infected individuals and healthy controls (HC) **f** Scatter plot shows correlation between % NK cell activation by anti-CD16 antibody (X axis) and Env-C stimulation (Y axis) (Spearman r test) The FcγRIIIa genotypes associated with HIV-specific ADCC response have been identified as Green dots (V/V genotype), Blue dots (Y/H genotype) and red dots (V/V-Y/H genotype)

The anti-HIV ADCC activity in the blood samples of the study participants was determined using NK cell

activation assay. The CD107a and IFN-γ expression by activated NK cells were used as surrogate markers for HIV-specific stimulation of NK cells in presence of autologous antibodies as described previously [4]. Briefly, the autologous NK cells from sodium-heparinized fresh whole blood from a study participant were stimulated with HIV-1C envelope peptides (Env-C) (obtained from NIH AIDS Reference Reagent Program; Catalogue No. 9499) at a final concentration of 1 μg/ml/peptide in the presence of brefeldin A (Sigma, 10 μg/ml), monensin (BD Biosciences, 0.68 μl/ml) and anti-CD107a APC Cy7 (clone H4A3; from Biolegend, San Diego, CA). The whole blood stimulated with purified anti-CD16 antibody (clone-3G8, 2.5 μg/ml) was used as a positive control and whole blood without stimulation was used as negative control. The plate was incubated for 5 h at 37 °C in 5% CO_2_ incubator. After incubation, CD3^−^ CD56^dim^ NK cells were gated and analyzed for the expression of intracellular IFN-γ and surface CD107a using fluorescent tagged antibodies [CD3 PerCP: (clone-SK7), CD56 PE-Cy7: (clone-HCD56), and CD107a APC-H7: (clone-H4A3) (all from Biolegend), and IFN-γ APC: (clone-B27) (BD Biosciences)]. The gating strategy is shown in Fig. 1a and d**;** (a) Lymphocytes were gated based on forward (FSC) and side scatter plot (SSC) and **(b)** NK cells were identified as CD3^-^CD56^dim^ cells and were assessed for either CD107a or IFNγ expression or dual expression. The percentage of activated NK cells as a marker of ADCC activity was represented as the sum of the percentage of activated NK cells, expressing only CD107a or IFN-γ, or both CD107a and IFN-γ (Fig. 1c, d) [3]. The response observed in negative (unstimulated whole blood) control was subtracted from respective Env-C stimulated response. The criteria for responders were i) the total percentage of CD107a and IFN-γ expressing (activated) NK cells was more than three times the percentage of activated NK cells from the negative control of the respective sample and ii) the individuals showing ADCC response above the mean plus two standard deviations (mean + 2SD) response observed in HIV uninfected healthy controls. The mean NK cell activation shown by HIV uninfected healthy individuals for HIV-1 envelope C was 0.64% (range: 0.00–2.95%) and no healthy control showed ADCC against HIV-1 Env C above the cut off value.

### Genotyping for FcγRIIIa receptor

Genomic DNA was extracted from peripheral blood using commercially available kit (QIAGEN, Inc., Valencia, CA) as per the manufacturer’s instructions and used for genotyping of FcγRIIIa receptor. Purity of eluted DNA was checked using Nanodrop (ND-1000). The purified DNA was further used for PCR amplification of *FCGR3A* gene using specific primers of exon 4 (Forward primer: 5′-ATGGCAAAGGCAGGAAGTAT − 3′, Reverse primer: 5′- CAACTTCCCAGTGTGATTGC-3′) as reported previously [14]. The amplification conditions were: Initial denaturation at 95 °C for 2 min, followed by 35 cycles at 95 °C for 30 s, 57 °C for 30 s, and 72 °C for 30 s, and final extension at 72 °C for 7 min. Amplified product of 218 bp was confirmed on 1% Agarose gel using 100 bp ladder. PCR amplicon was purified using high prep PCR clean up kit (Magbio Genomics Inc.). After the cleanup, PCR products were denatured to single stranded DNA using formamide. Purified DNA product was sequenced using BigDye Terminator® v3.1 cycle sequencing kit (Applied Biosystems, Foster City, CA) as per the manufacturer’s instructions. ABI PRISM® 310 Genetic Analyzer (Thermo Fisher Scientific) was used for sequencing of desired product and results obtained were aligned against reference sequence from UCSC genome browser (NM_001127593.1) using SeqScape v2.6 and its protein sequence then matched and analyzed for polymorphism using MEGA6 software.

### Statistical analysis

Statistical analyses were performed by using GraphPad Prism, version 5.0 (GraphPad software, San Diego, CA, USA). ANOVA-Bonferroni’s Correction test and two-tailed non parametric t-test (Mann Whitney) were used to determine the influence of genotypes on the magnitude of ADCC response. Genotype and allele frequencies of the FcγRIIIa within the study groups were compared using chi-square goodness-of-fit test or Fisher’s exact test as applicable. The correlation between the frequencies of anti-CD16 antibody activated NK cells by and Env-C stimulation was assessed by Spearman r test. *P* values < 0.05 were considered significant.

## Results

### Study population

Sixty three HIV infected and 67 HIV uninfected healthy individuals were enrolled in the study. The mean age of HIV infected individuals was 39 years (range 27–54), the mean CD4 count was 594 cells per μl (range 56–1583) and the mean viral load was 97,062 RNA copies per ml (range 0–1,475,011). The mean age of HC was 32 years (range 18–52). All the study participants were age matched and were of same ethnic background.

### HIV-specific ADCC responses

HIV-specific NK cell activation in presence of anti-HIV antibodies was used as a surrogate marker for HIV-specific ADCC response by number of studies [3, 4, 22–26]. Fifty seven percent (36/63) of HIV infected individuals showed Env-C specific NK cell (ADCC) response with a mean magnitude of 4.30% (range 0.0–18.80). No association of NK cell activation was observed with either CD4 count or viral loads. (Data not shown) The functionality of NK cells is known to be compromised in HIV infection [27, 28]. Therefore the analysis of NK cell activation after anti-CD16 stimulation in HIV infected and uninfected study participants was also carried out. It showed significantly lower anti-CD16 mediated NK cell activation in HIV infected individuals (mean: 13.16%; range 0.0–40.62) as compared to the response observed in uninfected controls (mean: 22.66%; range 0.42–43.01; *p* = 0.0018). **(**Fig. 1e) In HIV infected individuals, the anti-CD16 activated NK cell frequencies correlated positively with HIV-Env stimulated NK cell frequencies in presence of autologous anti-HIV antibodies indicating that the individuals showing ADCC responses had potent NK cells. (*p* < 0.0001, r^2^ = 0.36) **(**Fig. 1f**).**

### Identification of polymorphisms

Two polymorphisms were found in a 218 bp amplified PCR product. The earlier reported polymorphism at amino acid position 176 of FcγRIIIa receptor [12–14, 29, 30] was also observed in the Indian population. This polymorphism resulted in substitution of phenylalanine to valine at position 176 (V176F) (i.e. thymine (T) to guanine (G) transition) (NCBI dbSNP database accession number: ss2137543814). Another polymorphism was observed at nucleotide position 472 (at position 1,615,544,806 on chromosome 1/ 627 mRNA position) where transition from thymine (T) to cytosine (C) resulted in tyrosine to histidine at 158 amino acid position (Y158H) (NCBI dbSNP database accession number: ss2137543815).

### Genotype and allele frequencies in HIV infected and uninfected study participants

The genotypes (V/V, V/F, F/F, Y/Y & Y/H) and allele frequencies (V, F, Y & H) for the both polymorphisms are given in Table 1a, b. The analysis of genotype and allele frequencies showed significantly lower frequencies of Y/H (Y158H polymorphism) genotype and H allele in HIV infected study participants as compared to HCs. (Fisher’s exact test: *p* < 0.0001 & p < 0.0001 respectively; Table 1b) None of the study participants showed CC (H/H) genotype at 158 amino acid position in case of Y158H polymorphism.

**Table 1:** Frequency distribution of genotypes/allele in the study groups

**a. Genotype (VF variants)** **526 T/G (V176F)**	**HIV infected** ***N*** **= 63(%)**	**HC** ***N*** **= 67(%)**	**HIV infected vs HC**	
			**p value**	**OR (95% CI)**
TT(F/F)	19 (30.16)	23 (34.33)	1.00	Reference
TG(V/F)	36 (57.14)	34 (50.75)	0.56	0.78 (0.36–1.68)
GG(V/V)	08 (12.70)	10 (14.93)	1.00	1.03 (0.34–3.14)
**Allele (V176F)**	**HIV infected** ***N*** **= 126(%)**	**HC** ***N*** **= 134(%)**	**HIV infected vs HC**	
			**p value**	**OR (95% CI)**
T(F)	74 (58.73)	80 (59.70)	1.00	Reference
G(V)	52 (41.27)	54 (40.30)	0.90	1.04 (0.63–1.71)
**b. Genotype (YH variants)** **428 T/C (Y158H)**	**HIV infected** **N = 63(%)**	**HC** **N = 67(%)**	**HIV infected vs HC**	
			**p value**	**OR (95% CI)**
TT(Y/Y)	48 (76.19)	23 (34.33)	1.00	Reference
TC(Y/H)	15 (23.81)	44 (65.67)	**< 0.0001**	0.16 (0.07–0.35)
CC(H/H)	0 (0)	0 (0)	–	–
**Allele (Y158H)**	**HIV infected** **N = 126(%)**	**HC** **N = 134(%)**	**HIV infected vs HC**	
			**p value**	**OR (95% CI)**
T(Y)	111 (88.10)	90 (67.16)	1.00	Reference
C(H)	15 (11.90)	44 (32.84)	**< 0.0001**	3.62 (1.89–6.92)

a: Frequency distribution of V176F and Y158H polymorphisms in HIV infected vs HIV uninfected healthy control groups. Presence of TT (F/F) for TG (V/F), GG (V/V) genotypes, T for G allele was taken as reference group for statistical analysis

b: Frequency distribution of Y158H polymorphism in HIV infected vs healthy control groups. Presence of TT (Y/Y) for TC (Y/H), CC (H/H) genotypes, T for C allele was taken as reference group for statistical analysis

Fisher’s exact test was applied. N = number of subjects; (%) = frequency of genotypes/alleles, OR = Odds ratio; CI = Confidence Interval. The results with significance are shown in bold

### Env-C specific ADCC response and FcγRIIIa genotypes in HIV infected individuals

**Fig. 2:**
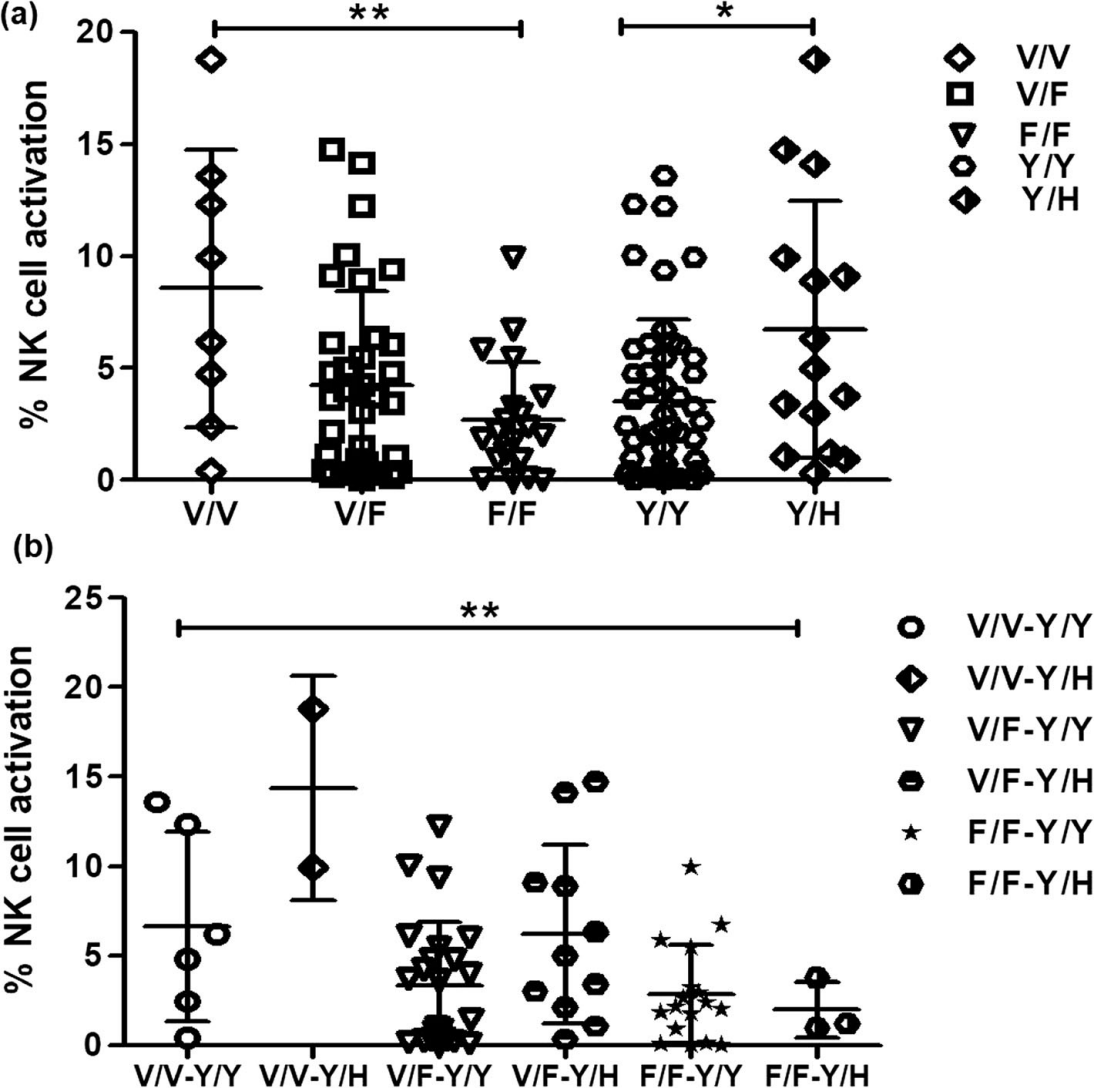
Variation in the magnitude of ADCC response with the different genotypes. Vertical scatter plot depicts **a** % NK cell activation in V/V-V/F-F/F & Y/Y-Y/H genotypes in HIV infected study group (Bonferroni’s multiple comparison of ANOVA for V/V-V/F-F/F genotypes and Mann Whitney t-test for Y and Y/H genotypes), The * indicates *p* < 0.05 and ** indicates *p* < 0.01 **b** % NK cell activation showed by the individuals showing different genotype combination (ANOVA-Bonferroni’s Correction test and ** indicates p < 0.01)

The magnitude of Env-C specific ADCC response (% activated NK cells after HIV-Env C stimulation in presence of autologous anti-HIV antibodies) was found to be significantly higher in individuals with V/V genotype (ANOVA Bonferroni’s Correction test) (*p* = 0.004; Fig. 2a) as compared to the individuals with either V/F or F/F genotype. Similarly in case of Y158H SNP, the individuals with the Y/H genotype showed significantly higher magnitude of ADCC responses as compared with the individuals with the Y/Y genotype (*p* = 0.032; Fig. 2a). Since both the polymorphisms showed significant influence on ADCC responses the combined effect of the dual polymorphism on the magnitude of ADCC response was assessed using ANOVA-Bonferroni’s Correction test and significantly higher magnitude of ADCC responses was observed in case of V/V-Y/H genotype combination though only two individuals showed this genotype combination (*p* = 0.001, Fig. 2b). In this study, when the association of anti-CD16 responses (baseline NK cell functionality) was compared with anti-HIV ADCC responses, results show that the individuals with the V/V–Y/H genotype (shown as red dots in Fig. 1f) have both higher anti-CD16 and anti HIV-1 ADCC responses.

However, significant difference was not found in the number of ADCC responders according to their genotypes (V/V or V/F or F/F or Y/Y or Y/H). Since the presence of one “V” had the highest frequency in our study population, the number of ADCC responders carrying at least one “V” allele was compared to those with the number of responders with FF allele however; there was no significant difference between the genotypes of ADCC responders vs non responders. (*p* > 0.05).

## Discussion

The genetic variants of FcγRIIIa receptor in HIV infected and uninfected Indian population and the association of these genotypes with the HIV-1 Env-C specific ADCC activity in infected individuals were analyzed in this study. Two SNPs at amino acid positions 176 and 158 resulting in replacement of phenylalanine to valine and tyrosine to histidine respectively were observed. The V176F polymorphism has already been reported in the various populations whereas to best of our knowledge, the Y158H polymorphism has been reported for the first time in Indian population. The frequency of FF genotype in the healthy controls in our study is 34.33% which was different from the frequencies reported in South Indian population [31]. This could be due to the differences in ethnicity since our study participants are mostly from Maharashtra, Western India. Similar differences in the V/V-V/F-F/F genotype frequencies have been reported in Japanese populations also [32–34]. Both these SNPs were found to be associated with higher Env-C specific ADCC response supporting previous observation of association of the V/V genotype in F176 V polymorphism with ADCC response [14, 21] and additionally showed association of the Y158H SNP with higher ADCC response. Previous studies have shown the association of high affinity CD16 receptor polymorphism with the clinical response of rituximab antibody in various lymphomas [35–37]. To best of our knowledge, this is the first report showing association of CD16 polymorphism with HIV specific ADCC response. The CD16 mediated ADCC activity is governed by NK cells [25]. NK cell are known to be functionally compromised in HIV infection [27, 28]. The association between the anti-CD16 mediated NK cell activation and HIV-1 Env C specific response in this study although expected, confirms that the ADCC response require binding to efficient NK cells and also underscore the importance of the affinity of the binding of Fc portion of antibody with FcγRIIIa (CD16a) receptor. Also it was observed that the individuals with the V/V and/or Y/H genotype have both higher anti-CD16 and anti HIV-1 ADCC responses hence it can be argued that the polymorphism in FcγRIIIa receptor might influence the binding of the Fc receptor of anti-HIV antibody and FcγRIIIa receptor on NK cells and thus in turn the magnitude of anti-HIV ADCC response. The FcγRIIIa receptor and Fc portion of the antibody bind to each other through a hydrogen bonding between the carbonyl group of Val 176 in FcγRIIIa and nitrogen of Gly236 in Fc [14]. It is possible that the strength of this binding is affected due to the structural difference caused by the amino acid substitution (Valine is substituted by Phenylalanine) which might have resulted in weak binding and less potent or no ADCC response. Similar possibility can be argued with the Y158H polymorphism. The ADCC could also be mediated by monocytes or neutrophils [22]. In this study, however the ADCC responses governed by these cells are not assessed. It might be possible that the individuals with Y/H or F/F or Y/H genotype could still show ADCC responses governed by these cells. However since the NK cells are the prime cells showing ADCC [4], the contribution of ADCC mediated by monocytes or neutrophils could be marginal.

The HIV disease progression has been shown to be associated with polymorphism in host genes such as HLA, CCR5, SDF-1, KIR, TRIM5α and the ABOBEC3 etc. [38–41]. The V176F polymorphism was not found to be associated with HIV-1 infection which was in the line with the previous reports showing absence of association between this polymorphism (V176F) and HIV infection [10, 18]. In contrast Poonia et al and others have reported that a high affinity allele V/V of *FCGR3A* gene is a risk factor for HIV-1 infection and disease progression [30, 42]. Racial differences resulting in different genetic makeup could be one of the likely reasons contributing to these contrasting reports.

The predominance of Y/Y genotype in HIV infected study participants in this study might indicate the probability of Y/Y genotype as a risk factor for HIV infection among Indians. The population variability of both these SNPs could be the major source for the variability and thus the association between these SNPs and HIV infection could be confirmed on larger sample size only when the true association could be assessed.

However, the observation that the Y/H genotype was associated with higher magnitude of HIV-1 specific ADCC response and association of Y/Y (not Y/H) genotype with HIV infection might raise an interesting possibility that the NK cell activation in HIV exposed but seronegative (ESN) individuals could be one of the factor for resistance to HIV acquisition. Indeed a report on ESN has shown risk of HIV acquisition with reduced NK cytotoxicity [43]. It would be interesting to study whether ESN also have Y/H genotype.

## Conclusion

The present study describes two new findings: (a) the positive influence of the V176F and Y158H polymorphisms on Env-C specific ADCC response, and (b) association of HIV infection and Y158H polymorphism. The ADCC response could be used as an important parameter of control over HIV and associated cancers where the monoclonal antibody based therapies might be used. In such situations, the genotyping carried out before such therapy will help in predicting the therapeutic efficiency. Additionally, modulation of broadly successful monoclonal antibodies by specific amino acid substitutions would be a useful approach to enhance FcγRIIIa binding and NK mediated ADCC response.

## Data Availability

The sequences are submitted to GenBank and are available in GenBank with accession number MG460484 - MG460567 and MH590780 - MH590835.

## Abbreviations

ADCC: Antibody dependent cell mediated cytotoxicity
Env-C: HIV-1C envelope peptides
ESN: HIV exposed but seronegative individuals
FSC: Forward Scatter
HCs: Healthy controls
HIV: Human Immunodeficiency Virus
NK cells: Natural killer cells
SNP: Single nucleotide polymorphism
SSC: Side Scatter
